# Can Double-Negative B Cells and Marginal Zone B Cells Have a Potential Impact on the Outcome of Kidney Transplantation?

**DOI:** 10.3390/jcm14103312

**Published:** 2025-05-09

**Authors:** Ariadni Fouza, Asimina Fylaktou, Maria Daoudaki, Persefoni Talimtzi, Anneta Tagkouta, Lampros Vagiotas, Georgios Katsanos, Georgios Tsoulfas, Nikolaos Antoniadis

**Affiliations:** 1 Department of Transplant Surgery, Center for Research and Innovation in Solid Organ Transplantation, School of Medicine, Faculty of Health Sciences, Aristotle University of Thessaloniki, 54124 Thessaloniki, Greece; ariadnefou@gmail.com (A.F.); lampisv@yahoo.gr (L.V.); katsanosg@auth.gr (G.K.); tsoulfasg@auth.gr (G.T.); nikanton@auth.gr (N.A.); 2 Department of Immunology, National Peripheral Histocompatibility Center, Hippokration General Hospital of Thessaloniki, 54642 Thessaloniki, Greece; fylaktoumina@gmail.com; 3 Laboratory of Biological Chemistry, School of Medicine, Faculty of Health Sciences, Aristotle University of Thessaloniki, 54124 Thessaloniki, Greece; taganneta@hotmail.com; 4 Department of Hygiene, Social-Preventive Medicine and Medical Statistics, School of Medicine, Faculty of Health Sciences, Aristotle University of Thessaloniki, 54124 Thessaloniki, Greece; persatalim@gmail.com

**Keywords:** double-negative B cells, marginal zone B cells, kidney transplantation, biomarkers of rejection

## Abstract

**Objectives/Background:** B lymphocytes are involved in both graft function and rejection. The role of double-negative (DN) and marginal zone B (MZB) lymphocytes in transplantation remains unclear. This study aims to investigate their role one year after transplant. **Methods:** The frequency and absolute numbers of DN and MZB cells were determined by flow cytometry before transplantation and at 3, 6 and 12 months after transplant. They were correlated with graft function and rejection. **Results:** Both the frequency and absolute number of MZB and DN cells increased 12 months after transplantation. Variations were observed in the populations studied at different time points. The observed decrease in the frequency of MZB lymphocytes in kidney recipients with rejection at 12 months, the end of follow-up, was associated with rejection episodes. On ROC curve analysis, a cut-off value of <20.6% could be a predictor of rejection risk in the first 12 months after transplantation (sensitivity 72.7%, specificity 69.6%). No relationship was found between the frequencies and absolute numbers of cell populations and graft function at any time point. **Conclusions:** The kinetics of B cells (DN and MZB) were determined over the course of 12 months after kidney transplantation. The frequency of MZ B cells was associated with rejection episodes.

## 1. Introduction

The combination of CD27 and immunoglobulin (Ig)D expression identifies four well-characterized B-cell subpopulations in human peripheral blood, namely, mature naïve B (NB) lymphocytes (CD19+CD27−IgD+); memory B lymphocytes without class switching, USM (CD19+CD27+IgD+); memory B lymphocytes with class switching, SM (CD19+CD27+IgD−); and double-negative (DN) B lymphocytes (CD19+CD27−IgD−). CD27, a member of the tumor necrosis factor receptor (TNFR) superfamily, serves as a marker for memory B lymphocytes distinguishing memory from naive B lymphocytes [1]. DN B lymphocytes (identified as memory cells due to mutated IgV genes) are present at a very low frequency at birth but represent approximately 5% of all peripheral B lymphocytes in healthy adults [2]. A high degree of similarity has been observed between the BCR repertoires of CD27− and CD27+ memory B lymphocytes, with these CD27− cells being identified as potential precursors of CD27+ memory B lymphocytes [3]. DN B lymphocytes represent a heterogeneous population composed of four subsets based on the relative expression of CD21 and CD11c [4], with different origins and functions [5,6]. DN B lymphocytes bear properties analogous to those of SM B cells with regard to telomere length and the expression of different isotypes of immunoglobulin [5], and there is a possibility that they may develop from SM B cells [7,8]. Sanz et al. [4] characterized and studied three of the four subsets, which were designated DN1, DN2 and DN3 [7,8]. DN cells have been observed at high frequencies in autoimmune diseases such as arthritis and systemic lupus erythematosus (SLE), with DN1 and DN2 subsets being observed in SLE [7], and in chronic infections with hepatitis C virus, human immunodeficiency virus (HIV) or malaria [9,10]. Furthermore, Frasca et al. [11] have suggested that DN B lymphocytes are proinflammatory cells due to their age-related characteristics.

In the context of renal transplantation, Shuller et al. [12] found an increase in the frequency of the DN population one year after transplantation but no change in absolute numbers. Conversely, a previous study of ours showed no change in the frequency of DN cells six months after transplantation in recipients with stable graft function [13].

Another distinctive memory B-lymphocyte lineage that has been identified is the MZ B lymphocyte [14]. The immunophenotype of this particular cell is CD19+CD27+IgM+IgD+. These account for almost 15–20% of splenic B lymphocytes and approximately 15% of peripheral blood B lymphocytes [15,16]. They are innate-like B cells, and they express poly-reactive BCRs and function as a primary line of defense, eradicating pathogens, particularly encapsulated bacteria and apoptotic cellular debris. Following antigen uptake, MZ B lymphocytes possess the potential to differentiate into plasmablasts, a process that may or may not be dependent on T lymphocytes. Furthermore, MZ B lymphocytes have been observed to be particularly sensitive to toll-like receptor (TLR) stimulation, which has been shown to enhance their primary antibody response to blood-borne pathogens and T-independent antigens [15]. Their involvement in both host protection and autoimmunity is well documented [15], but data on MZ B cells in transplantation are limited. Asano et al. [17] showed that intrarenal B cells in human kidney allografts, which are associated with a poor prognosis in kidney transplantation, are innate-like cells on the basis of transcriptional studies.

In a preclinical model of cardiac allograft transplantation, the inhibition of CD40-CD40L co-stimulatory molecule signaling selectively enhances anti-inflammatory IL-10 expression in MZ B cells, resulting in immune tolerance [18]. More recently, MZ B cells were shown to contribute to the development of DSA in response to heart and kidney transplantation in a murine transplantation model [19].

In the field of clinical transplantation, Zhuang Q et al. [20] observed that MZB cells decreased both in number and frequency one year after kidney transplantation compared to levels in dialysis patients and healthy volunteers. Furthermore, San Segundo D and colleagues [21] discovered that a reduction in MZB cells in the peripheral blood of kidney transplant patients was associated with an increased risk of kidney graft rejection.

The current literature on the contribution of marginal zone cells and double-negative B cells in renal transplant recipients is limited. The primary objective of this prospective study was to obtain kinetic data on the two populations (DN and MZ B cells) over a 12-month period after transplantation, with data collection occurring at 3-month intervals, and to evaluate any differences between pre-transplant levels and those at different study times, as well as to determine the association between the two populations and clinical settings. Additionally, this study also investigated how various parameters affecting transplant outcome affected both populations and assessed the impact of these parameters on graft rejection.

This study was conducted using flow cytometry at three-month intervals over a one-year period in a cohort of 71 transplant patients from a single institution.

## 2. Methods

### 2.1. Study Patients

The study cohort comprised seventy-one recipients who had undergone ABO-compatible kidney transplantation. This study was conducted as a prospective, observational, single-center investigation. Of the kidney recipients, 64 had previously undergone dialysis prior to transplantation, while 7 recipients were enrolled preemptively (without having undergone dialysis) for kidney transplantation.

Patients undergoing renal transplantation (TN) were assessed at the time of transplantation (T0) and followed prospectively for a period of 12 months, with assessments at 3 months (T3), 6 months (T6) and 12 months (T12) after transplantation.

#### 2.1.1. Inclusion Criteria

Eligible participants were adults between the ages of 18 and 60 years who had been regular attendees at the transplant center outpatient clinic for at least 2 years prior to transplantation.

#### 2.1.2. Exclusion Criteria

Patients were excluded from the study if they had a documented history of serious recent infections, recent malignancy, active autoimmune or inflammatory disease, hematological disorders or immunosuppressive treatment during the 12 months prior to kidney transplantation.

Moreover, the same decision was applied to individuals experiencing an acute deterioration of renal function of unknown etiology and to patients who did not have their renal function monitored for a period of less than two years.

Recipients of transplants from deceased donors with cardiac arrest were also excluded from the study, as were patients who did not comply with treatment instructions.

### 2.2. Study Schedule

All recipients of a kidney transplant were considered eligible for the study on the basis of the above-mentioned inclusion criteria. Patients were included if they were on dialysis before transplantation or preemptively (11%). The day of transplantation was considered the day of enrollment and defined as T0. Demographic and clinical information (Table 1), as well as details on medical history, primary disease and treatment, were extracted from the patients’ medical records.

Blood samples were collected for laboratory and immunological evaluation (which included DN and MZ B cells) at four distinct time points: prior to transplantation and before the commencement of any immunosuppressive therapy (T0) and at 3 months (T3), 6 months (T6) and 12 months (T12) after transplantation.

This enabled comparisons to be made between different time points before and after transplantation.

Renal function, medications and potential side effects were recorded. Renal function was evaluated using eGFR with the CKD-EPI (2021 update, (Chronic Kidney Disease Epidemiology Collaboration) formula by estimating the glomerular filtration rate (e-GFR).

### 2.3. Ethical Approval and Consent to Participate

The study was conducted in accordance with the ethical regulations and the principles of the Declaration of Helsinki, as well as with the approval of the Institutional Review Board of the Medical School of the Aristotle University of Thessaloniki (protocol code 4356, date of approval 26 January 2021). Written informed consent was obtained from each patient.

### 2.4. Immunosuppressive Regimen

The maintenance immunosuppressive regimen followed the protocol established by the Transplantation Centre. It comprised a combination of corticosteroids, a calcineurin inhibitor (tacrolimus) and an anti-proliferative agent (mycophenolate mofetil). Induction therapy consisted of either an anti-CD25 antibody (basiliximab) or antithymocyte globulin. Of the patients, 88.7% (63 patients) received basiliximab.

### 2.5. Flow Cytometry of B-Cell Subpopulations

To identify DN and MZ B cells, the following monoclonal antibodies, conjugated to fluorochromes, were employed:

- Anti-CD19 PC5.5 clone J3-119, Beckman Coulter, Beckman Coulter, Inc., Sykesville, MD, USA;
- Anti-CD27 PE-Dylight 594 clone LT27, EXBIO, Praha SA, Czech Republic;
- Anti-CD45-PC7 clone J33 Beckman Coulter, Beckman Coulter, Inc., Sykesville, MD, USA;
- Anti-IgD clone IA6-2 Thermo Scientific LSG, Lagoas Park, Porto Salvo, Portugal;
- Anti-IgM PE clone, SA-DA4 Beckman Coulter, Beckman Coulter, Inc., Sykesville, MD, USA.

Flow cytometric analysis was conducted using a Navios EX flow cytometer (Beckman Coulter, Sykesville, MD, USA). Duplicate, dead or dying cells were excluded by plotting forward scatter height versus forward scatter area, and lymphocytes were identified based on their forward and side scatter characteristics. All experiments involved analysis via forward scatter/side scatter gating on lymphocytes, with the gating process performed manually by a single operator.

The complete circulating population of B cells was recognized as CD19+. Moreover, DN B cells were defined as CD19+CD27−IgD− and memory MZ B cells as CD19+CD27+IgD+IgM+. Frequencies were determined relative to CD19+.

The results for the analysis of DN and MZ B cells are presented in Figure S1 in the Supplementary Data.

### 2.6. Statistics

Descriptive statistics were calculated using the mean and standard deviation or median and interquartile range (IQR) for quantitative variables, while frequencies and percentages were used for qualitative variables. The Shapiro–Wilk test for normality was used for quantitative variables. Friedman’s ANOVA for non-parametric variables was utilized to examine changes in subpopulations between 0, 3, 6 and 12 months.

Subsequent to Friedman, a post hoc test was utilized to ascertain which subgroups exhibited significant differences. Univariate and multivariate linear logistic regression was performed to investigate the effect of different factors on different cell populations. Factors were recipient age, donor type (deceased/living), CTI, DGF and dialysis vintage. Parameters with a *p*-value of less than 0.20 in the univariate analysis were included in the final model. The chi-squared test and Wilcoxon rank sum test were used to compare the characteristics of kidney transplant recipients and cell populations between renal transplant recipients with and without episodes of rejection. Receiver operator characteristic (ROC) curves were used to determine the predictive value of MZ B cells for rejection episodes. The statistical significance level was set at *p* ≤ 0.05. Statistical analysis was performed using R statistical software (version 4.3).

## 3. Results

### 3.1. Patient Characteristics

A total of 90 patients were enrolled in the study; however, 19 of these patients withdrew from the study during the follow-up period. The final number of patients included in the study was seventy-one, and a detailed description of their characteristics can be found in Table 1. The analysis of the dataset revealed that eight patients had missing data in their medical records, two patients had died, two patients had experienced a relapse of the primary disease, three patients had contracted an infection during the follow-up period and four patients had withdrawn their consent from the study.

### 3.2. Differences in DN and MZB B-Cell Populations at Different Study Time Points: DN and MZ B Cells Underwent Changes During the 12-Month Follow-Up Period

**Figure 1:**
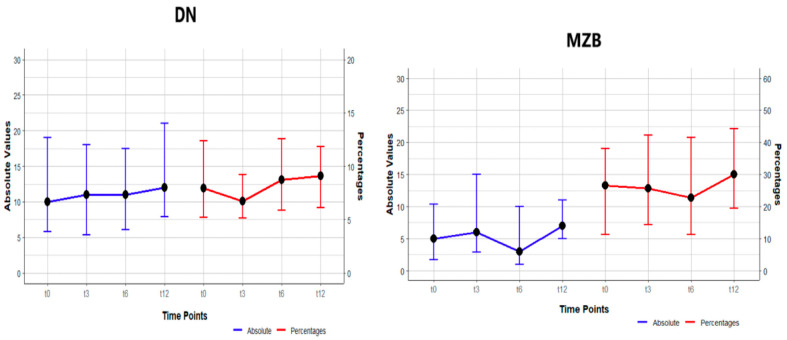
Changes in frequency (percentages) and absolute numbers in DN and MZ B cells at study points T0, T3, T6, T12. The values are presented as median (IQR).

A rise in the frequency of double-negative memory B lymphocytes was observed at the conclusion of the study (*p* = 0.004). It is noteworthy that the value of *p* = 0.004 for double-negative B cells was not regarded as indicating a statistically significant increase, as it did not demonstrate significance in the post hoc comparison. Frequencies were examined at all designated time points, revealing non-statistically significant differences between them, including a decrease from pre-transplant levels of 11.9 (7.8, 18.6) to 10.1 (7.7, 13.8) at 3 months after transplant, followed by a subsequent increase to 13.1 (8.8, 18.82) at 6 months following transplant, after which values remained stable. A study of the marginal zone lymphocytes (MZB) indicated a non-significant difference, with a slight decline in frequency observed at T3 and T6. With regard to absolute counts, a rise was observed in double-negative cells, although this rise did not achieve statistical significance. Marginal B-cell absolute counts demonstrated an initial increase at T3, followed by a subsequent decline at T6 and subsequent increase at T12. It is noteworthy that *p* = 0.005 for marginal zone B cells was not regarded as a statistically significant difference, as it did not demonstrate significance in the post hoc comparison, as shown in Figure 1 and Table 2.

### 3.3. How the DN and MZ Β Cell Populations Were Affected by Different Factors That Influence the Outcome of Kidney Transplantation

The effect of different factors influencing graft function and thus the outcome of the transplantation on the frequencies and the corresponding absolute numbers of B-lymphocyte subpopulations at T3, T6 and T12 was studied.

These factors were related to the donor (deceased or living), to the recipient (age, dialysis vintage), to the transplant process (duration of cold ischemia time, CIT) and to the post-operative complication of delayed graft function, DGF.

The univariate regression analysis revealed a tendency toward an association between dialysis vintage and the frequency of IgD⁻CD27⁻ B cells, as show in Table 3. However, this association did not attain statistical significance (*p* = 0.05, 95% CI: −0.04 to 0.02; Table 3).

A multivariate analysis could not be conducted for the frequency of DN and MZ B cells, since the criteria used were not met. This is outlined in full in Section 2, and the reasons for its application are discussed there.

In addition, a univariate regression model was utilized to analyze the impact of dialysis vintage, recipient age, donor type, cold ischemia time and DGF on the absolute number of both DN and MZ B cells. A number of factors were found to have a significant impact on the absolute number of IgD-CD27-B cells, including dialysis vintage, age of the recipient, type of donor and cold ischemia time. With regard to MZ B cells, DGF was the sole significant factor influencing their absolute numbers. In the multivariate model, no statistically significant factors were identified for either of the B-cell subpopulations under evaluation, as shown in Table 4.

### 3.4. MZ B Cells in Relation to the Prediction of Rejection

During the 12-month follow-up period, 11 patients experienced graft rejection, as diagnosed by graft biopsy. It should be noted that the collection of protocol biopsies is not standard practice at this center; rather, this is conducted exclusively when clinical indications suggest it is necessary. As the time of biopsy did not correspond with the time of immunophenotyping, the patients experiencing rejection during the 12-month follow-up period were analyzed collectively.

The study population was divided into two groups: one consisting of the 11 patients with rejection and the other consisting of 60 patients free of rejection. The characteristics of the patients are described in Table 5 below.

The hypothesis was that the pre-transplant immunological profile, as determined by the immunophenotype of DN and MZ B lymphocytes, would influence the outcome of the transplant. To this end, a comparison was made between the two B-lymphocyte subpopulations in the two groups in terms of both frequency and absolute numbers at T0. A similar comparison was made at the end of the twelve-month follow-up period at T12. The results revealed a difference between the group with rejection episodes and the group without rejection at both time points for both populations, as shown in Table 6 and Table 7.

At T0, both the DN and MZB populations demonstrated a decline in frequency and absolute numbers, those the difference did not reach statistical significance. For DN cells, the rejection group showed a frequency of 9% (7, 15) versus 12% (8, 19) in the rejection-free group, where *p* = 0.5. In terms of absolute numbers, the rejection group showed a value of 9 (6, 18) versus 10 (6, 19) in the rejection-free group, where *p* = 0.8. For MZ B cells, the frequency was 17 (9, 27) in the rejection group versus 31 (9, 39) in the rejection-free group, *p* = 0.5, and the absolute number was 4.2 (1.8, 6.3) in the rejection group versus 5.2 (1.1, 11.8) in the rejection-free group, *p* = 0.7.

At T12, the DN population exhibited a non-significant increase in frequency and a non-significant decrease in absolute numbers in the rejection group in comparison to values in the rejection-free group. With regard to the frequency of MZ B cells, a decrease was observed in the rejection group compared to that in the rejection-free group, where *p* = 0.064, a value that approached statistical significance (see Table 7). The absolute numbers demonstrated a non-significant increase, with 7 (5, 11) in the rejection-free group versus 9 (6, 10) in the rejection group, *p* > 0.9.

**Figure 2:**
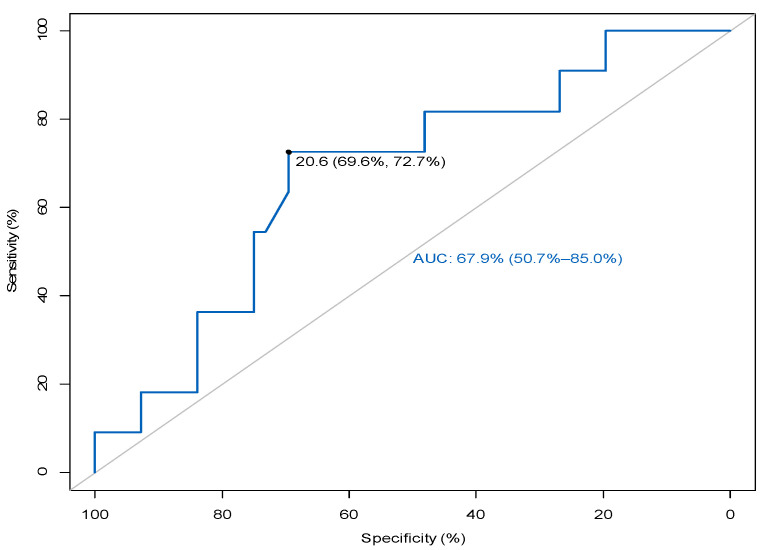
ROC curve analysis to differentiate patients with rejection episodes from those who were free of rejection.

Given the observed trend toward significance in the frequency of MZ B cells, the investigation focused on determining the potential of this population as a predictor of rejection. The relationship between MZ B cells and episodes of rejection was highlighted by constructing a receiver operating characteristic (ROC) curve and calculating the area under the curve (AUC). The ROC analysis determined the optimal cut-off value for MZ B cells and showed that an MZB frequency of <20.6% could be a possible predictor of patients at risk of rejection in the first 12 months after transplantation, with a sensitivity of 72.7% and a specificity of 69.6%, as shown in Figure 2.

### 3.5. Frequencies and Absolute Numbers of MZ and DN B Cells in Relation to Graft Function at Different Time Points During Study Follow-Up

A correlation analysis was conducted between the frequencies and absolute numbers of the immunological populations mentioned above and GFR values (an assessment of kidney function). At time points T3, T6 and T12, DN and MZB cells did not show a significant correlation with eGFR in terms of either frequency or absolute numbers, as shown in Table S1, Supplementary Data.

Despite the absence of a statistically significant correlation between the frequency and absolute numbers of DN and MZB cells and graft function, a further investigation was conducted into the potential correlation between cell populations and graft function. To this end, patients were divided into two groups according to their eGFR at all study time points. Two groups were thus produced, with Group 1 comprising patients with an eGFR > 60 mL/min/1.73 m^2^, while Group 2 consisted of individuals with an eGFR < 60. It is noteworthy that the number of patients in each group varied at different study time points, as shown in Table S2, Supplementary Data. The results revealed no statistically significant correlation between DN and MZB cell populations when patients were grouped based on their eGFR. Consequently, it can be deduced that the two populations did not exert a positive influence on renal function during the 12-month follow-up period.

### 3.6. Effect of Induction Immune Therapy on DN and MZ B Cells at Different Time Points

It was found that the induction therapy did not have any effect on the frequency or the absolute number of the two populations of B cells, DN and MZ, at the different time points of the study, as shown in Tables S3 and S4, Supplementary Data.

## 4. Discussion

In recent years, the field of clinical transplantation has seen a significant focus on graft acceptance, with research efforts concentrating on the role of memory B cells and the regulation of alloimmune responses through B regulatory (Breg) and T regulatory (Treg) cells. However, research attention has been very limited regarding the study of atypical DN memory B cells and marginal B cells.

The present study demonstrated that the frequency of double-negative memory cells exhibited an increase at the conclusion of the 12-month follow-up. This finding is consistent with the results reported in a study conducted by Shuller et al. [12]. However, the observed increase in absolute numbers is contrary to the results of the aforementioned study [12], which documented no change. In addition, an increase in frequency and a significant increase in the absolute number of marginal zone B cells were observed, although these increases did not reach statistical significance at 12 months. No differences in frequency were observed at T3 and T6, which is consistent with the findings of Alfrano et al. [22]. However, Zhuang et al. [20] reported a decrease in the frequency of marginal zone B lymphocytes one year after transplantation.

With regard to DN, the role of these cells in transplantation remains to be elucidated. However, in the context of autoimmune diseases, there is mounting evidence suggesting their pathogenic role, as exemplified by lupus erythematosus. By definition, this population of memory cells is fully differentiated and does not proliferate but produces proinflammatory mediators. The presence of these cells fosters an inflammatory microenvironment that facilitates their differentiation into plasmablasts and the subsequent secretion of alloantibodies, a process that may potentially cause AMR [23].

Factors such as age of the recipient, dialysis vintage, time of cold ischemia and type of donor (deceased or living), which influence the outcome of transplantation, affect the absolute number of DN B cells. DGF affects the absolute number of MZB cells. Data on the effects of the above factors on an individual basis for each subpopulation are scarce to non-existent in the literature, indicating the novelty of this study.

In a correlation analysis to ascertain the relationship between renal function and DN and MZB populations, the results indicated that there was no statistically significant correlation between the frequency and absolute number of either population at three, six or twelve months following transplantation. This suggests that the populations under investigation did not appear to correlate with graft function.

The frequency of the MZB cell population at T12 decreased within the group of recipients who experienced an episode of rejection. This finding is consistent with those reported by San Segundo et al. [21], who also identified a decrease in the frequency of MZB cells.

Stranavova et al. [24] presented the findings of a study on three kidney recipients who underwent preemptive transplantation. Two of the subjects who experienced acute rejection had elevated pre-transplant frequencies of MZB cells compared to those in recipients on dialysis. In a subsequent analysis of a larger number of preemptive and non-preemptive recipients, recipients with higher pre-transplant MZB frequencies showed a trend toward an increased rejection rate during the first year after transplantation, albeit not statistically significant [24].

In contrast to the findings of Stranavova et al. [24], our estimation of the pre-transplant MZB cell frequency in preemptive kidney recipients was lower than that in the recipients on dialysis [25]. Notably, one of the eleven patients who experienced a rejection episode was a preemptive recipient.

In order to advance our understanding of the function of MZ B cells in the context of kidney transplantation, this study explored the potential of MZ B-cell frequency as a non-invasive marker for the accurate and timely diagnosis of rejection episodes. The rationale for selecting this particular cell population was based on the observed differences in frequencies between patients with and without rejection, which approached statistical significance at T12. Given their function as memory cells [26], MZ B cells have been hypothesized to play a pivotal role in the outcome of transplantation due to their ability to be activated following antigen exposure. In addition, it was hypothesized that MZB cells would have an effect on graft function, as they have been found to act as a reservoir of alloreactive B lymphocytes activated by allogeneic antigens in preclinical models [27].

The analysis of the receiver operating characteristic (ROC) curve used to determine the prognosis of acute rejection in kidney transplantation based on the frequency of B-lymphocyte subpopulations yielded results indicating that in cases where the frequency of MZB cells is less than 20.6% (with a sensitivity of 72.7% and a specificity of 69.6%), the patient is considered at risk of experiencing a rejection episode within 12 months of transplantation.

San Segundo et al. [21] previously reported a reduction in MZB cells in the peripheral blood of kidney transplant recipients, which is consistent with the results of the current study. Nevertheless, they found that a MZ B-cell frequency of less than 3.55% was associated with an increased risk of kidney graft rejection, with their results obtained based on biopsy-diagnosed rejection. The frequency of the MZ B population in the two groups studied, a group with rejection and a group free of rejection, showed numerically lower values than those observed in our study. This is due to the fact that the researchers used different markers to identify MZ B cells, and the time points in the study were different, making it difficult to compare the cut-off values of the two studies.

Although these results are encouraging and add to the body of knowledge in this field, further studies are needed to confirm the results obtained and to define cut-off values. Only then will it be possible to propose the MZ B-cell subpopulation as a possible biomarker for acute rejection in kidney transplantation. Furthermore, it is essential to expand the sample size by testing larger cohorts of patients from multicenter trials. These samples should be characterized using the same MZ B-cell markers, and the results should be replicated prior to a rejection episode diagnosed by biopsy.

The present study revealed a lower frequency of circulating MZ B cells in the group of kidney recipients who experienced rejection. However, the underlying cause of this phenomenon remains to be elucidated. One hypothesis is that this reduction may be due to the migration of these cells into the graft. This possibility remains unconfirmed due to the lack of data on this population from renal biopsies. However, recent evidence from preclinical models suggests that this may be a plausible scenario.

A comprehensive study is currently underway with the aim of comparing the results of circulating MZ B cells with those of intrarenal MZ B cells. This approach is expected to provide further insight into the above hypothesis and to propose MZ B cells as a possible rejection risk marker in kidney recipients one year after transplantation.

Moreover, while this study was not originally designed to discuss the role of T-cell subpopulations in renal allograft acceptance, a number of such studies have been performed, but the results obtained so far are limited to a sample of 40 patients, without being representative of the entire study population. In accordance with the regulatory properties of MZB (IL-10 production) [15], correlation analyses were conducted between MZB cells and Tregs (CD4+CD25+FOXP3+) at T0 and T12. However, no association was identified between the two populations at either of the two time points. Further investigation will be conducted on different T-cell subpopulations in a larger cohort of patients owing to the ongoing research in this field.

The present study is not without limitations. In addition to the need to increase the sample size through collaboration with transplant centers, both nationally and internationally, this study did not characterize the functionally distinct DN subpopulations. Such an analysis may provide more information on the association of DN cells with graft function and renal transplant outcome.

## 5. Conclusions

The present study demonstrated alterations in DN and MZ B-cell populations one year following KT. The temporal progression of these populations at three-month intervals has not been previously documented in the current literature. Further studies are required to ascertain the role of DN cells in kidney transplantation. The findings of this study highlight the association between the frequency of MZ B cells and the rejection of kidney grafts, underscoring the potential for this cell population to serve as biomarker for rejection.

## Figures and Tables

**Table 1:** Demographic and clinical characteristics of kidney transplant recipients enrolled in the study.

Study Sample	N: 71
**Characteristics of recipients**	
Sex	Female: 20/Male: 51 28.17/71.83%
Age in years	48.5 (39, 60)
Type of donors	Deceased/brain death: 50 (70%) Living: 21 (30%)
Preemptive recipients	7 (9.86%)
**Dialysis patient candidates for transplantation**	
Type of dialysis	HD: 64 (81%) CAPD: 7 (19%)
Duration of dialysis (months)	87 (34–127)
**Distribution of underlying kidney disease**	
Polycystic kidney disease	14 (19.7%)
Primary glomerulonephritis IgA nephropathy Membranous nephropathy Focal segmental glomerulosclerosis Membranoproliferative glomerulonephritis	12 (17%) 5 (7%) 3 (4.25%) 2 (2.83%) 2 (2.83%)
Reflux nephropathy	6 (8.4%)
Diabetes mellitus	6 (8.4%)
Nephrosclerosis/hypertension	8 (11.25%)
Urinary tract infections/stones	5 (7%)
Other	12 (17%)
Unknown	8 (11.25%)
**Information on transplantation, graft function**	
Delayed graft function	Yes: 12 (24%) No: 38 (76%)
Cold ischemia time (hours)	19.2 (4.6)
eGFR (mL/min/1.73 m^2^)	52 (36–89)
Recipients with rejection	11 (15.5%)
**Induction therapy**	
Basiliximab, N (%)	63 (88.7%)
Anti-thymocyte globulin, N(%)	8 (11.3%)

**Table 2:** Changes in the frequency and absolute numbers of the DN and MZ B-cell populations at T0, T3, T6 and T12.

	T0 ^1^	T3 ^1^	T6 ^1^	T12 ^1^	*p* ^2^	Post Hoc ^3^ Comparison
**%Double negative, (CD19+IgD−CD27),** **DN**	11.9 (7.8, 18.6)	10.1 (7.7, 13.8)	13.1 (8.8, 18.8)	13.6 (9.2, 17.7)	0.004	**ns**
**#Double negative** **(CD19+IgD−CD27−),** **DN**	10 (5.8, 19)	11 (5.3, 18)	11 (6.1, 17.5)	12 (7.9, 21)	ns	**ns**
**%Marginal zone B cells (CD19+IgD+IgM+CD27+), MZB**	26.6 (11.2, 38.1)	25.6 (14.4, 42.2)	22.8 (11.3, 41.6)	30 (19.5, 44.3)	ns	**ns**
**#(CD19+IgD+IgM+CD27+), MZB**	5 (1.7, 10.4)	6 (2.8, 15)	3 (1, 10)	7 (5, 11)	0.005	**ns**

Median ^1^ (IQR); Friedman ^2^ test; Wilcoxon ^3^ signed-rank test.

**Table 3:** Results of the univariate analysis of the frequencies of DN and MZ B cells in relation to the age of recipients, the type of donor, cold ischemia time, delayed graft function and dialysis vintage. β: beta coefficient; *p*-value, values of *p* ≤ 0.05 were considered statistically significant; N: number of participants in the study. The number of participants differs in the dialysis vintage parameter because the preemptive candidates were not included.

	Univariate Regression				
Frequency of Cell Population				95% Confidence Interval	
	β	Ν	*p*	Lower	Upper
**Double negative, DN**					
Age of the recipient	−0.04	71	0.5	−0.17	0.09
Type of donor (deceased/living)	1.6	71	0.3	−1.8	5
Cold ischemia time, CIT	−0.04	71	0.7	−0.20	0.13
Delayed graft function, DGF	2.8	71	0.093	−0.49	6.1
Dialysis vintage	−0.01	64	0.05	−0.04	0.02
**Marginal zone B cells, MZB**					
Age of the recipient	0.07	71	0.7	−0.27	0.42
Type of donor (deceased/living)	−1.2	71	0.8	−10	8
Cold ischemia time, CIT	−0.19	71	0.5	−0.28	0.6
DGF	−4.1	71	0.4	−13	4.7
Dialysis vintage	0.04	64	0.4	−0.04	0.12

**Table 4:** Results of univariate and multivariate regression analyses of the absolute numbers of DN and MZ B cells in relation to the age of recipients, the type of donor, cold ischemia time, delayed graft function and dialysis vintage. β: beta coefficient; *p*-value, values of *p* ≤ 0.05 were considered statistically significant; N: number of participants in the study. The number of participants differs in the dialysis vintage parameter because the preemptive candidates were not included.

	Univariate Regression					Multivariate Regression			
Absolute Numbers of Cells				95% Confidence Interval				95% Confidence Interval	
	β	Ν	*p*	Lower	Upper	β	*p*	Lower	Upper
**Double negative, DN**									
Age of the recipient	−0.47	71	0.01	−0.47	−0.82	−0.27	0.3	−0.75	0.21
Type of donor (deceased/living)	11	71	0.023	1.5	20	5.9	0.6	−15	27
Cold ischemia time, CIT	−0.49	71	0.031	−0.94	−0.05	−0.1	0.8	−1	0.84
Delayed graft function, DGF	−2.0	71	0.7	−12	7.5				
Dialysis vintage	−0.12	64	0.005	−0.12	−0.21	−0.05	0.4	−0.17	0.07
**Marginal zone B cells, MZB**									
Age of the recipient	−0.19	71	0.079	−0.19	0.02	−0.11	0.3	−0.35	0.12
Type of donor (deceased/living)	5.6	71	0.052	−0.05	11	6.4	0.3	−5	18
Cold ischemia time, CIT	−0.20	71	0.2	−0.47	0.07	0.2	0.5	−0.34	0.74
DGF	−5.4	71	0.047	−11	−0.07	−4.4	0.12	−10	1.2
Dialysis vintage	0.00	64	>0.9	−0.04	0.03				

**Table 5:** Characteristics of kidney transplant recipients who experienced episodes of rejection and those who were free of rejection. *p*-value * by Wilcoxon rank sum test.

	Kidney Transplant Recipients (N: 71)	Recipients Experiencing Rejection (N: 11)	Recipients Free of Rejection (N: 60)	*p*-Value *
**Age of recipients**	49 (40, 57)	49 (38.25, 57)	48 (43, 52)	0.968
**Sex (female/male)**	19/52	3/8	16/44	1.000
**Donor type** **(deceased/living)**	50/21	7/4	43/17	0.721
**DGF**	21/71	6/11	15/60	0.072

**Table 6:** Changes in frequency and absolute numbers of DN and MZ B cells in renal transplant recipients with and without episodes of rejection at T0.

Frequency and Absolute Number of Cells at Pre-Transplant Time (T0)	Rejection		*p*-Value Wilcoxon Rank Sum Test
	Rejection Free, (N = 60) Median (IQR)	Rejection, (N = 11) Median (IQR)	
% DN	12 (8, 19)	9 (7, 15)	0.5
# DN	10 (6, 19)	9 (6, 18)	0.8
% MZB	31 (9, 39)	17 (9, 27)	0.5
# MZB	5.2 (1.1, 11.8)	4.2 (1.8, 6.3)	0.7

**Table 7:** Changes in frequency and absolute numbers of DN and MZ B cells in renal transplant recipients with and without episodes of rejection at T12.

Cell Population at 12 Months Post-Transplant	Rejection		*p*-Value Wilcoxon Rank Sum Test
	Rejection Free, N = 60 Median (IQR)	Rejection, N = 11 Median (IQR)	
% DN	12.9 (8.7, 17.9)	14.4 (12.7, 17.6)	0.2
# DN	12 (8, 21)	11 (9, 25)	0.7
% MZB	31 (20, 45)	20 (13, 26)	0.064
# MZB	7 (5, 11)	9 (6, 10)	>0.9

## Data Availability

Upon request, the corresponding author can provide the datasets used and analyzed in this study.

## Supplementary Materials

The following supporting information can be downloaded at: https://www.mdpi.com/article/10.3390/jcm14103312/s1.
