# Supplementary material for: Can Double-Negative B Cells and Marginal Zone B Cells Have a Potential Impact on the Outcome of Kidney Transplantation?
Source: J Clin Med. 2025 May 9;14(10):3312. doi: 10.3390/jcm14103312 (PMC12112073; doi:10.3390/jcm14103312)
Supplement: Supplementary file 1 [file jcm-14-03312-s001.zip › jcm-3612487-supplementary.pdf]

Table S1: Correlation coefficients of the association of DN (frequency and absolute numbers), and MZB (frequency and absolute numbers) with the mean or median eGFR value at T3,T6, T12. Values of r were calculated using Spearman's rank correlation (*) and Pearson's rank correlation (**) tests.

**Cell Populations**		**eGFR** **T3**	**T6**	**T12**
DN %	r	0.46**	-0.135*	0.09*
	p-value	0.1	0.49	0.436
# DN Cells/µL	r	0.41*	0.005**	0.16
	p-value	0.18	0.74	0.30
MZB %	r	0.72*	-0.409**	0.350*
	p-value	0.33	0. 16	0.39
# MZB Cells/µL	r	0.9**	0.23*	0.64**
	p-value	0.42	0.63	0.337

Table S2: Correlation coefficients of the association of DN (frequency and absolute numbers), and MZB (frequency and absolute numbers) with the mean or median eGFR value > 60 and eGFR value <60 at T3,T6, T12. Values of r were calculated using Spearman's rank correlation (*) and Pearson's rank correlation (**) tests.

**Cell Populations**		**T3 n:31**	**T3 n:40**	**T6 n:38**	**T6 n:33**	**T12 n: 47**	**T12 n:24**
		**eGFR>60**	**eGFR<60**	**eGFR>60**	**eGFR<60**	**eGFR>60**	**eGFR<60**
%DN	r	-0.240*	0.167*	-0.135**	0.136**	0.874*	0. 64*
	p-value	0.271	0.284	0.419	0.536	0.11	0.68
#DN Cells/µL	r	0.118**	0.117*	0.005*	-0.106**	0.128*	0.36*
	p-value	0.591	0.456	0.974	0.630	0.130	0.42
%MZB	r	0.067*	-0.017*	-0.033*	0.18*	0.061*	0.32*
	p-value	0.760	0.914	0.34	0.11	0.524	0.38
#MZB Cells/µL	r	0.114**	0.075*	-0.063*	0.32**	0.705*	0.52**
	p-value	0.605	0.632	0.76	0.5	0.47	0.68

Table S3:

Changes in frequency and absolute numbers of DN and MZ B cells in renal transplant recipients who are on a basiliximab therapy N: 63 compared to total number of kidney transplant recipients at T3, T6 and T12.

Cell Populations	T3	T3 Basiliximab	p	T6	T6 Basiliximab	p	T12	T12 Basiliximab	p
%DN	10.1 (7.7, 13.8)	8.8 (7.9, 13.1)	ns	13.1 (8.8, 18.8)	12 (9.1, 19.2)	ns	13.6 (9.2, 17.7)	14 (10, 16.9)	ns
#DN Cells/μL	11 (5.3, 18)	10.1 (6.3, 17)	ns	11 (6.1, 17.5)	12 (6.8, 16.2)	ns	12 (7.9, 21)	12.5 (6.5, 20.8)	ns
%MZB	25.6 (14.4, 42.2)	24.9 (14, 43.4)	ns	13.1 (8.8, 18.8)	12 (9.1, 17.2)	ns	30 (19.5, 44.3)	28.9 (20, 42.1)	ns
#MZB Cells/μL	6 (2.8, 15)	5.7 (4.1, 13.7)	ns	3 (1, 10)	3.2 (2.8, 8.8)	ns	7 (5, 11)	5.8 (6, 12)	ns

Table S4:

Changes in frequency and absolute numbers of DN and MZ B cells in renal transplant recipients who are on a ATG therapy N: 8 compared to total number of kidney transplant recipients at T3, T6 and T12.
ATG: Anti-thymocyte globulin, ND:not determined, ns :non significant

Cell Populations	T3	T3 ATG	p	T6	T6 ATG	p	T12	T12 ATG	p
%DN	10.1 (7.7, 13.8)	ND		13.1 (8.8, 18,8)	12,9 (8.1, 18.2)	ns	13.6 (9.2, 17.7)	14.8 (10, 18.1)	ns
#DN Cells/μL	11 (5.3, 18)	ND	ns	11 (6.1, 17.5)	11.1 (7.2, 18.1)	ns	12 (7.9, 21)	11.5 (7.5, 20.1)	ns
%MZB	25.6 (14.4, 42.2)	ND	ns	13.1 (8.8, 18.8)	12.7 (9.4, 17.8)	ns	30 (19.5, 44.3)	29 (19, 43.2)	ns
#MZB Cells/μL	6 (2.8, 15)	ND	ns	3 (1, 10)	3 (2.6, 9.1)	ns	7 (5, 11)	6.8 (6,6, 10.9)	ns

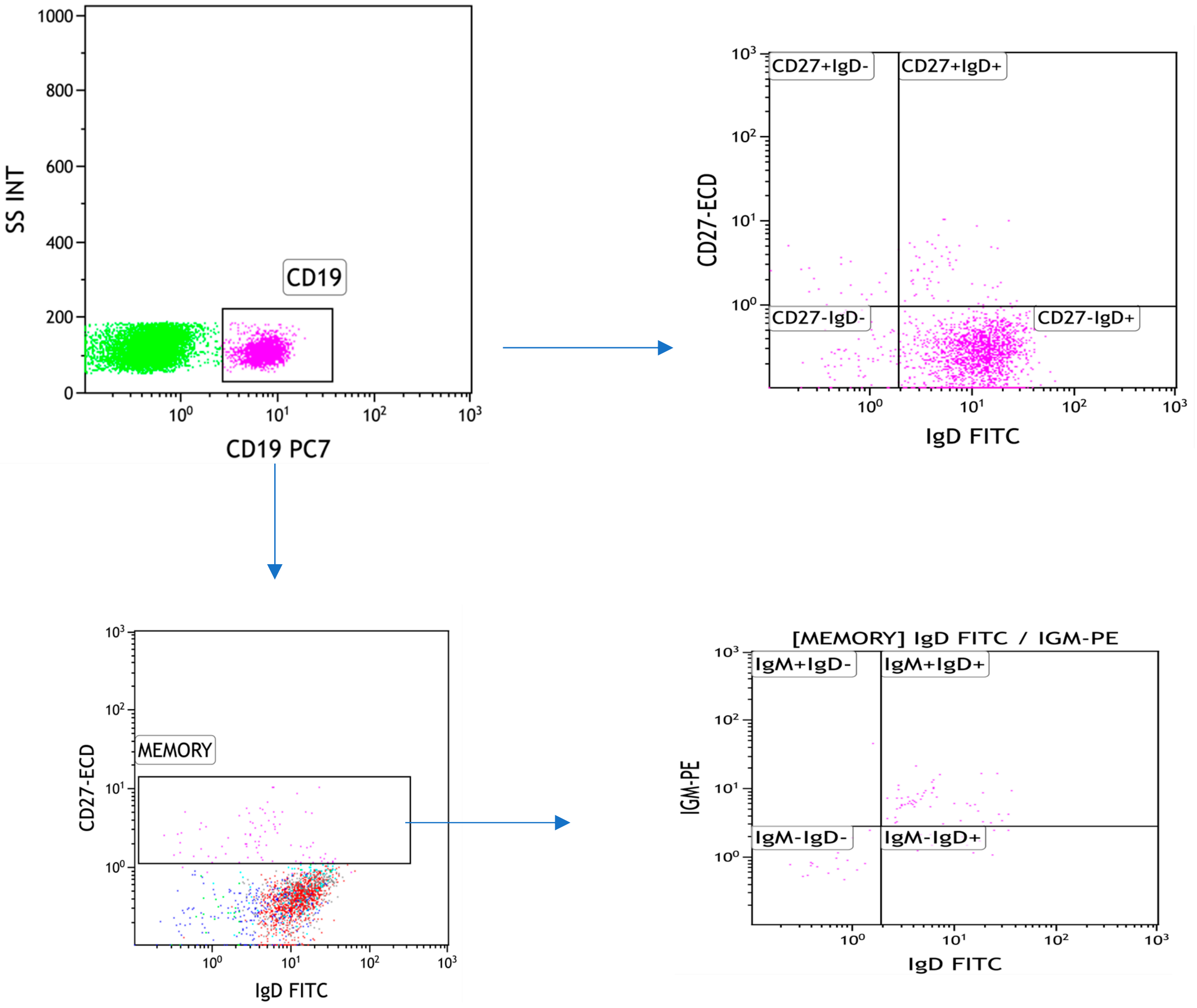

Figure S1:
Representative plots of DN (CD19+CD27-IgD-) and MZ (CD19+CD27+IgD+IgM+) B cell populations. Using the memory gate CD19+CD27+IgD-, MZ B cells were identified as CD19+CD27+IgD+IgG+.
